# Reverse genetics rescue of sylvatic dengue viruses

**DOI:** 10.1128/jvi.00450-25

**Published:** 2025-06-04

**Authors:** Paul Gendler, Arturo Barbachano-Guerrero, Samuel D. Chappell, Oshani C. Ratnayake, Samantha M. Pinto, Rushika Perera, Sara L. Sawyer

**Affiliations:** 1 Department of Molecular, Cellular, and Developmental Biology, University of Colorado Boulder 315558, Boulder, Colorado, USA; 2 BioFrontiers Institute, University of Colorado Boulder 458481, Boulder, Colorado, USA; 3 Department of Microbiology, Immunology, and Pathology, Colorado State University 164597 , Fort Collins, Colorado, USA; Emory University School of Medicine, Atlanta, Georgia, USA

**Keywords:** dengue virus, reverse genetics

## Abstract

There are an unknown number of sylvatic (“sylvan” = “of the forest”) dengue viruses currently being sustained by nonhuman primates and mosquitoes in the forests of Africa and Asia. Humans are regularly infected with these viruses, occasionally resulting in small epidemics. One barrier to understanding sylvatic dengue virus biology is the scarcity of stocks available for study. While the full-length genome sequences of 28 sylvatic viruses exist on GenBank, accessible infectious stocks exist for only a handful of these. One way to overcome this obstacle is to rescue infectious virus stocks using reverse genetics. However, reverse genetic engineering of dengue viruses is notoriously difficult. Here, we optimize a reverse genetics method specifically for the rescue of sylvatic dengue virus stocks from sequence data. The key to our approach was the observation that mosquito cells, rather than mammalian cells, must be used to launch sylvatic dengue virus replication from assembled genomes. We demonstrate the success of this technique by rescuing seven sylvatic dengue viruses. With this unique collection, we then characterized the relative fitness of each virus strain on human, monkey, and mosquito cells. While mosquito cells are universally permissive for the growth of sylvatic dengue viruses, some sylvatic dengue virus strains showed significantly better replication in human and monkey cells than others. These sylvatic dengue virus strains may have a greater potential for human adaptation.

**IMPORTANCE:**

Given the enormous burden of the four human dengue viruses, which emerged from the sylvatic dengue virus reservoir, it is important that we consider the possibility of a new dengue virus emerging into the human population. Nonhuman primate species in Asia and Africa are suspected to be the natural reservoir hosts for sylvatic dengue viruses. Occasionally, these sylvatic dengue viruses infect humans, although there are few stocks of these viruses available for study in the lab. Here, we optimize a reverse genetics technique for sylvatic dengue viruses, and we rescue stocks of seven strains. With this method, theoretically, any sylvatic dengue virus sequence deposited on GenBank can be transformed into a high-titer infectious virus stock.

## INTRODUCTION

There are four human dengue viruses (Family *Flaviviridae*, species *Orthoflavivirus denguei*) named dengue virus 1–4 (DENV1-4) (1). These viruses are found in tropical and subtropical regions, where nearly half of the world’s population lives (2). Each year, up to 10% of people in dengue-endemic regions become infected (3). Dengue viruses are transmitted by mosquitoes of the *Aedes* genus. As climate change alters the range of these mosquitoes, the range of dengue viruses is also changing (4). In 2023, historic dengue outbreaks occurred worldwide. In Southeast Asia, Bangladesh experienced its largest outbreak ever, with over 200,000 recorded cases and nearly 1,000 deaths (5). In Peru, recorded dengue cases exceeded the previous 5-year average by 10-fold (6). Burkina Faso in West Africa saw over 500 deaths due to dengue, a staggering increase from the 18 and 15 deaths reported in 2017 and 2016, respectively (7). Although most dengue cases are asymptomatic, severe infections can lead to dengue hemorrhagic fever, dengue shock syndrome, and even death (8). While vaccine development has been a slow and arduous process, two dengue virus vaccines have been recently approved for use (9, 10). As dengue epidemics intensify, these vaccines offer hope in controlling future outbreaks.

**Fig 1:**
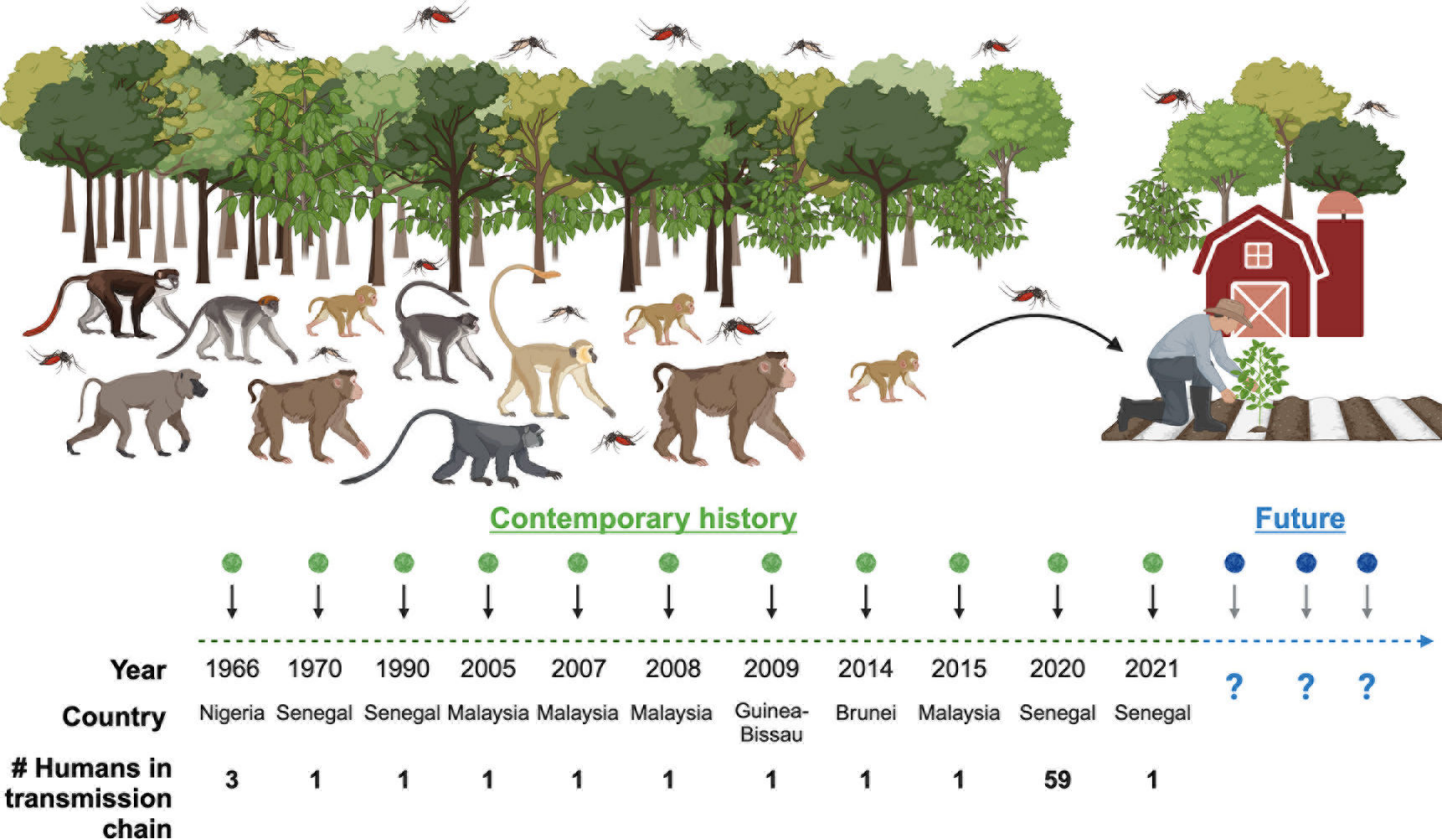
The history of human infections with sylvatic dengue viruses. This figure summarizes the history of sylvatic dengue virus infections in humans, as reported in the literature from the 1960s to today. In 1966, three closely related strains of sylvatic dengue virus (strains IBH11234, IBH11208, and IBH11664) were isolated from humans in Nigeria (12, 18). In 1970 and 1990, strains DakHD10674 and DakHD76395 were isolated from humans in Senegal (12, 19). In 2005, strain P72-1244 was isolated from a patient with dengue fever in Malaysia, a strain that was first isolated from a sentinel monkey in 1972 (12, 20). In 2007 and 2008, two additional strains, DKE-121 and DKD811, were isolated from patients in Malaysia (21). In 2009, strain EEB-17 was isolated from a traveler returning from Guinea Bissau (22). In 2014 and 2015, strains Brun2014 and DSab2015 were isolated from travelers returning from Brunei and Malaysia, respectively (23, 24). In 2020, a small outbreak of sylvatic dengue virus occurred in Senegal, with 59 infections being documented and three strains sequenced (strains SH356683, SH356692, and SH356702) (17). A year later, an additional sylvatic dengue virus infection was confirmed in a man in Senegal (25).

Given the burden of the four human dengue viruses and the long and challenging path to developing vaccines against them, it is crucial to consider the possibility of a new dengue virus emerging into the human population. In Africa and Asia, “sylvatic” (“sylvan” = “of the forest”) dengue viruses are transmitted between wild primates by arboreal *Aedes* mosquitos (11–14). Phylogenetic analyses suggest that sylvatic dengue viruses spilled over into humans on four independent occasions, giving rise to the four human dengue viruses (15, 16). While these enormously consequential spillovers occurred long before recorded medical history, recent reports of humans infected with sylvatic dengue viruses highlight the ongoing risk posed by these viruses. Since the 1960s, sporadic human infections with sylvatic dengue viruses have been continuously documented (Fig. 1). In most cases, only a single human infection was documented. However, occasional small outbreaks have occurred, such as in Kedougou, Senegal, in 2020 (17). It is important to underscore that the cases in Fig. 1 only represent those that evoked medical scrutiny and, further, documentation in the literature. Therefore, far more people are probably infected by sylvatic dengue viruses than what is represented here.

It remains unknown what biological properties differentiate the sylvatic viruses that can infect humans and spread between them. The virology and immunology of sylvatic dengue viruses are unstudied except for a few papers (26–31). One hypothesis is that some sylvatic viruses are more pre-adapted to the human host than others, but as of yet, there is no support for this hypothesis, and data remain extremely limited on how different strains of sylvatic dengue viruses replicate in human cells (27). Addressing questions like these is difficult because there are few readily available sylvatic dengue virus stocks. In fact, infectious stocks of only four strains of sylvatic dengue virus are available through the main repository in the U.S., the Biodefense and Emerging Infections Research Resources Repository (BEI Resources). Limited accessibility to virus stocks is also the case with other emerging viruses, highlighting the need for innovative techniques to synthesize and propagate these viruses in the lab (32, 33).

One solution to this problem is to generate virus stocks from sequence data using reverse genetics. Reverse genetics requires two key steps: assembling the viral genome from sequence data and launching an infectious virus in cell culture. In the launch step, the assembled viral genome is transfected into human or other eukaryotic cells that will support transcription of viral RNA, translation of viral proteins, and virion assembly and release. Genome assembly for orthoflaviviruses is known to be very challenging due to genomic instability and toxicity to bacterial hosts (34). Orthoflavivirus genomes are believed to encode cryptic promoters that drive the synthesis of viral products that are toxic to bacteria, making traditional cloning and assembly of these genomes in bacteria difficult. While various strategies have been developed to mitigate this problem, most remain inefficient, labor-intensive, or technically challenging (35–37). In contrast, bacterial-free approaches allow genome assembly without the instability associated with bacterial hosts (for a relevant review, see reference 38). Among these, circular polymerase extension reaction (CPER) has emerged as a leading technique for assembling positive sense RNA virus genomes *in vitro* (39–41). CPER has been successfully applied to various orthoflaviviruses (42–46), including in the launch of a Zika virus (47) and the launch of 20 diverse dengue virus strains by Tamura et al. (19).

Our initial attempts to assemble and launch sylvatic dengue virus genomes using CPER were unsuccessful. Here, we optimize both the genome assembly and launch of sylvatic dengue viruses. The key to our success was the observation that mosquito cells, rather than mammalian cells, must be used to launch sylvatic dengue virus replication from CPER-assembled genomes. We used this optimized protocol to rescue stocks of seven strains of sylvatic dengue virus. With this unique collection, we then characterized the relative fitness of each virus strain on human, monkey, and mosquito cells. First, we found that mosquito cells are universally permissive for the launch and subsequent propagation of sylvatic dengue viruses. Second, we found that some sylvatic dengue virus strains showed significantly better replication in human and monkey cells than others.

## RESULTS

### *In vitro* assembly of a sylvatic dengue virus genome (strain DakArA1247)

**Fig 2:**
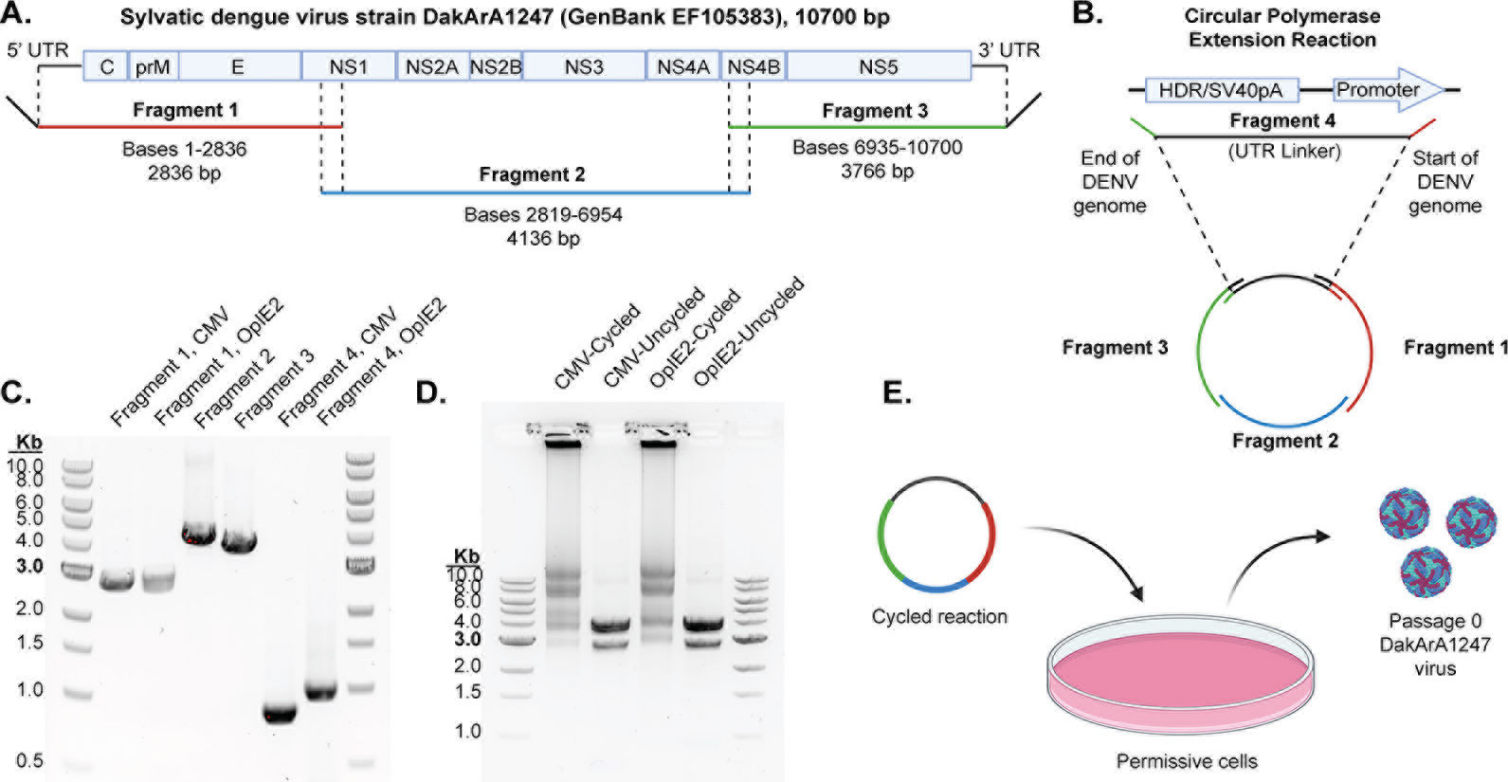
*In vitro* assembly of a sylvatic dengue virus genome (strain DakArA1247). (**A**) The sequence of sylvatic dengue strain DakArA1247 was obtained from GenBank. From this, three overlapping DNA fragments were designed and synthesized by a commercial vendor. Diagonal lines indicate overhangs added by primers during fragment amplification. (**B**) In addition, two alternate “UTR-linker” sequences (fragment 4) were synthesized, differing only by the promoter they contain. Diagonal lines indicate overhangs added by primers during fragment amplification. The CPER assembles all four products into circularized form. (**C**) Upon receipt of synthetic DNA fragments 1–4, each fragment was amplified by PCR. Fragment 1 was amplified with a forward primer that added an overhang sequence complementary to the end of the promoter region of fragment 4; thus, two promoters are indicated in the fragment 1 lane. Also, the two versions of fragment 4 are shown. (**D**) PCR products of the four fragments were combined at equimolar ratios and cycled in a thermocycler to perform the CPER. Two bands are evident in the uncycled control: fragments 2 (4.1 kb) and 3 (3.8 kb) are similar sizes and presumably run together as the upper band, and the lower band is fragment 1 (2.8 kb). The linker fragment (~800 or 1,000 bp depending on the promoter) is too dilute to see. (**E**) The product of the CPER is then directly transfected into permissive cells to launch virus replication.

We performed a proof-of-principle study on a sylvatic dengue virus strain called DakArA1247, which was isolated from an *Aedes taylori* mosquito trapped in the Ivory Coast (12). Using a commercial vendor, we synthesized the DakArA1247 genome as three overlapping DNA fragments (Fig. 2A). We also synthesized a fourth “UTR linker” fragment (Fig. 2B). This UTR linker fragment contains a promoter that will ultimately sit upstream of the viral genome and drive transcription. The UTR linker fragment also contains a hepatitis delta virus ribozyme/simian virus 40 polyadenylation signal (HDR/SV40pA) that will sit downstream of the viral genome. The simian virus 40 polyadenylation signal facilitates transcriptional termination and stabilizes the newly transcribed RNA (48), while the hepatitis delta virus ribozyme sequence autocleaves to detach itself and the simian virus 40 polyadenylation signal from the infectious positive-sense viral transcript (49). We constructed two versions of the UTR linker: one contains the cytomegalovirus (CMV) promoter that is active in mammalian cells, and the other contains the baculovirus OpIE2 promoter which is active in insect cells (50). Promoter sequences can be found in Fig. S1.

Upon receipt of these DNA fragments, we amplified each individual fragment by PCR (Fig. 2C). Because fragment 4 shared no overlap with fragments 1 and 3, fragment 4 was amplified with forward and reverse primers that added overhang sequences complementary to the end of the dengue virus genome and the start of the dengue virus genome, respectively. Primer sequences used to amplify each fragment can be found in Tables S1 and S2. Each fragment was then purified after PCR, and the final product concentration was determined.

Next, PCR products (fragments 1–4) were combined at equimolar ratios with a polymerase and put into a thermocycler to perform the CPER assembly (51, 52). During CPER, overlapping fragments are joined together, forming a circular DNA molecule (Fig. 2B). However, because the UTR linker overlaps with both the start and end of the viral genome, we can also expect to see head-to-tail joining of full-length genomes in either circular or linear form. Indeed, we observed a variety of CPER products: an ~11 kb product, which represents the length of all four fragments joined together; a series of bands between 3 and 10 kb, which represent unassembled fragments and assemblies of just two or three fragments; and smears with large amounts of DNA remaining in the well, which likely represent a range of large concatemerized assemblies of genomes (Fig. 2D). It remains unclear whether the fragment at ~11 kb, the large concatemerized multiples of this fragment, or both, contribute to launching the virus in the next step. Nonetheless, the whole reaction mix (unpurified) is then directly transfected into permissive cells (Fig. 2E), as described in the next section.

### Identification of cell lines that can launch sylvatic dengue virus replication from *in vitro* assembled genomes

**Fig 3:**
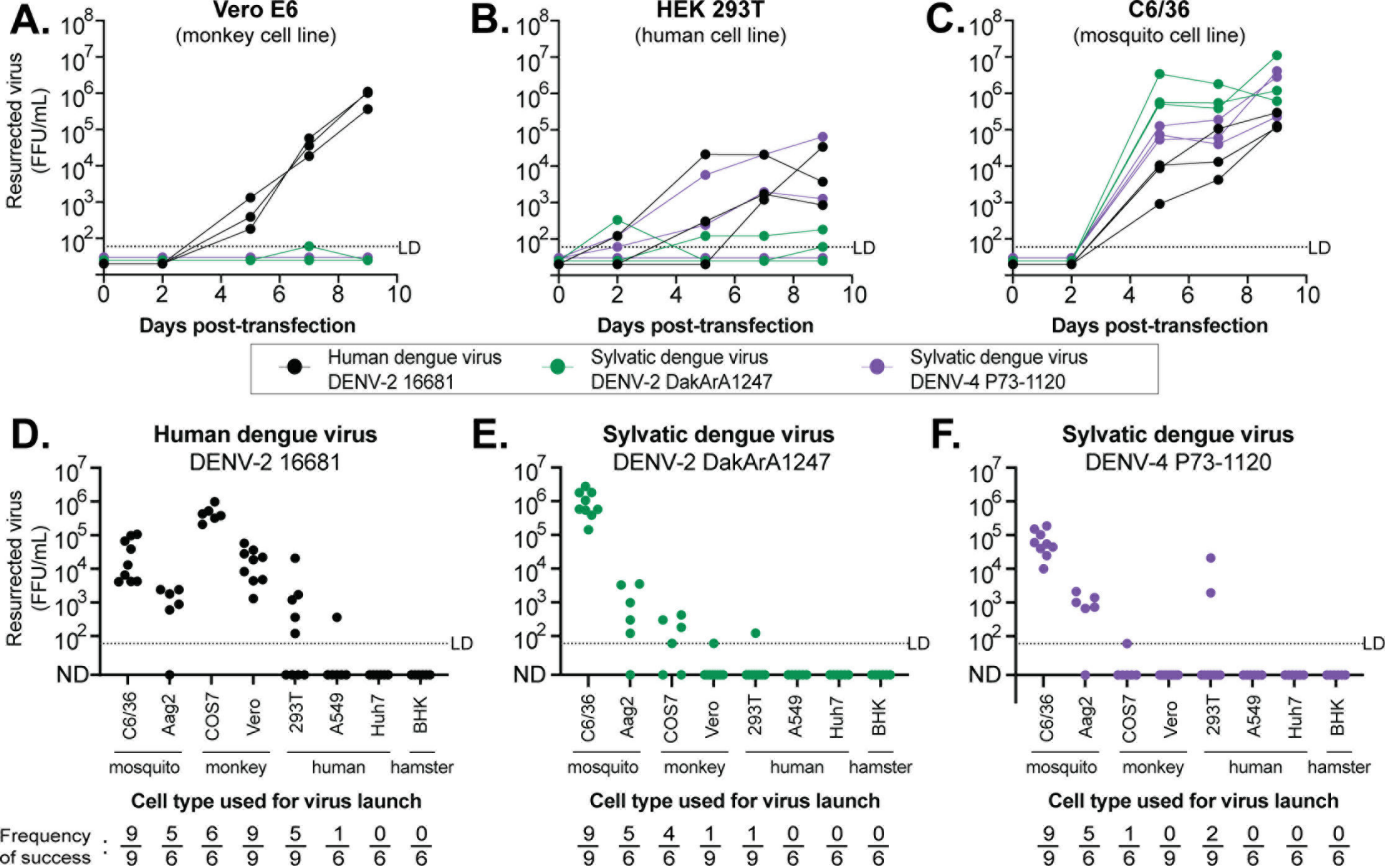
Identification of cell lines that can launch sylvatic dengue virus replication from *in vitro* assembled genomes. (**A–C**) Dengue virus genomes (legend) were assembled by CPER, with either (A and B) the mammalian CMV promoter or (**C**) the insect OpIE2 promoter. Assembly products were transfected into either (**A**) Vero E6, (**B**) HEK 293T, or (**C**) C6/36 cells. Supernatants were collected at the indicated days post-transfection. The infectious virus in the supernatant at each time was determined by a focus forming unit (FFU) assay. Each line represents one of the three biological replicates. The dotted line represents the limit of detection (LD); points underneath the LD were arbitrarily assigned a value for visualization purposes. (**D–F**) CPER-assembled dengue virus genomes (top of graphs) were transfected into eight different cell lines. Supernatants were collected 7 days post-transfection, and virus titers were obtained by FFU assay. This entire process was repeated 6–9 times for each cell line, and each dot represents an independent experiment. The dotted line represents the limit of detection; points underneath were plotted at ND (not detected). The frequency of success for launching a virus in each cell type (bottom of graphs) was calculated by dividing the number of experiments that yielded at least one infectious virus (by FFU assay) by the total number of individual experiments attempted.

Once the viral genome is assembled with CPER, it is transfected into human or other eukaryotic cells that will support transcription of viral RNA, translation of viral proteins, and virion assembly and release. Two cell lines commonly used for launching human dengue viruses are African green monkey Vero E6 and human HEK-293T (37, 52–56). To test if these cells support the launch of sylvatic dengue viruses, we transfected them with genomes assembled by CPER for a human dengue virus strain (16681) and two sylvatic strains (DakArA1247 and P73-1120). We analyzed virus production over time with a focus-forming unit (FFU) assay. In Vero E6 cells, the human dengue virus, but neither of the sylvatic dengue viruses, successfully launched (Fig. 3A). This was unexpected because Vero E6 cells are known for their impaired interferon response and general permissiveness to viruses (57–59). In HEK-293T cells, both human 16681 and sylvatic P73-1120 viruses launched, but the sylvatic DakArA1247 strain did not (Fig. 3B). Since all dengue viruses, including sylvatic dengue viruses, are transmitted by mosquitos, we hypothesized that mosquito cells may be more effective for launching sylvatic DakArA1247. Mosquito cells have previously been used to launch multiple orthoflaviviruses, such as Japanese encephalitis virus, yellow fever virus, and Zika virus (60, 61). Therefore, we again CPER-assembled genomes of the human strain 16681 and sylvatic strains DakArA1247 and P73-1120, this time using the insect OpIE2 promoter. CPER products were transfected into the *Aedes albopictus* C6/36 mosquito cell line. All three dengue viruses launched in C6/36 cells and reached titers higher than those observed in the tested mammalian cells (Fig. 3C).

Recognizing the importance of selecting the optimal cell type for virus launch, we next explored a broader collection of cell lines. We transfected CPER-assembled genomes for all three viruses into eight different cell lines, then enumerated infectious virus produced at 7 days post-transfection (Y axis in Fig. 3D through F). We performed each experiment 6–9 times so that we could also determine the frequency of successful viral launch in each cell line (bottom of graphs in Fig. 3D through F). Both mosquito cell lines tested, C6/36 (*Aedes albopictus*) and Aag2 (*Aedes aegypti*), supported the launch of all three viruses. Compared to the mammalian cell lines, more virus was produced (Y axis), and the launch was more often successful (bottom of graph) in mosquito cell lines. On occasion, the mammalian cell lines tested supported the launch of sylvatic dengue viruses, but we were never able to propagate these viruses further, likely due to the low amount of virus produced. Viruses were produced to higher titers in C6/36 cells than in Aag2 cells. This is likely because C6/36 cells are deficient in RNA interference (RNAi) signaling (62).

### A sylvatic dengue virus (DakArA1247) rescued by this reverse genetics method exhibits similar properties to an existing virus stock obtained from BEI Resources

**Fig 4:**
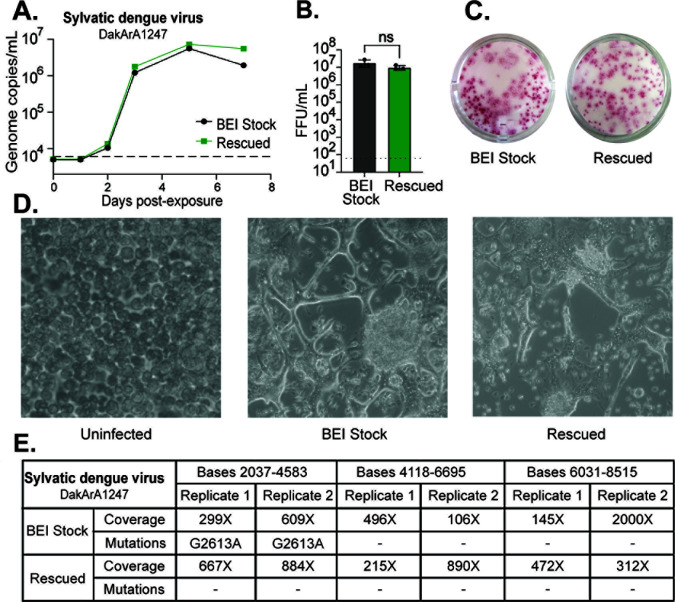
A sylvatic dengue virus (DakArA1247) rescued by this reverse genetics method exhibits similar properties to an existing stock obtained from BEI Resources. (**A**) C6/36 cells were exposed to stocks of DakArA1247 that were either obtained from BEI Resources or rescued via the method herein. The multiplicity of infection was 0.1, and cell supernatants were collected at 0, 1, 2, 3, 5, and 7 days post-exposure, from which viral RNA was extracted and analyzed by RT-qPCR. Synthetic dengue genomic RNA was used to generate a standard curve of genome copies per milliliter, from which Y-axis values were derived. The dotted line represents the limit of quantification. (**B**) Supernatant was collected 5 days post-exposure, and the concentration of infectious virus was determined by FFU assay. The dotted line represents the limit of detection. (**C**) Representative images of foci obtained by testing supernatant 5 days post-exposure by FFU assay. (**D**) Representative images of cytopathic effects observed on C6/36 cells 5 days post-exposure. (**E**) cDNA was generated from extracted viral RNA from each virus. PCR amplicons spanning bases 2,037–4,583, 4,118–6,695, and 6,031–8,515 of the DakArA1247 genome were amplified from cDNA in two independent reactions and sequenced. Sequencing coverage and identified mutations are listed.

We wished to compare a sylvatic dengue virus launched by this reverse genetics method to an existing stock of the same strain. We launched the CPER-assembled genome of sylvatic dengue virus strain DakArA1247 in C6/36 mosquito cells and also obtained a stock of this same strain from BEI Resources (Cat# NR-12221). C6/36 mosquito cells were then infected with each of these virus stocks at a multiplicity of infection of 0.1. The amount of viral RNA in the supernatant increased at similar rates for both strains (Fig. 4A). Additionally, both strains produced similar levels of infectious virions by 5 days (Fig. 4B) and formed foci with similar morphology (Fig. 4C). We had previously observed that this strain, DakArA1247, induces a distinctive cytopathic effect in C6/36 mosquito cells during replication. This effect is characterized by the loss of the cells’ round shape and the formation of elongated syncytia, which was consistently produced by both the rescued and BEI stocks (Fig. 4D).

To further compare the two virus stocks, we analyzed their sequences against the GenBank reference sequence (EF105383). Fragments spanning most of the viral genome (bases 2,037–8,515) were amplified by PCR and sequenced using Oxford Nanopore sequencing. We found that the rescued virus had no mutations compared to the GenBank reference sequence, while the BEI stock virus had a synonymous mutation in the NS1 gene (Fig. 4E). We can speculate that this mutation may have arisen during the passage of the BEI stock. This analysis highlights a virtue of reverse genetics: virus stocks generated are identical to the original genome sequence each time they are produced, ensuring greater reproducibility and preventing the accumulation of mutations during passage.

### Generation of a panel of infectious stocks of diverse sylvatic dengue viruses

Next, we aimed to rescue a diverse range of sylvatic dengue viruses. We identified 28 full-length genome sequences of sylvatic dengue virus strains on GenBank (Table 1). From these, we selected a phylogenetically diverse set of seven strains (Fig. S2). The full genome of each virus was assembled using CPER, with the insect promoter, the products of which were transfected into C6/36 mosquito cells to launch the production of virions. Supernatants were collected 7 days post-transfection and directly transferred to a 15 cm plate of C6/36 cells to allow for the propagation of “passage 0” virus, for 7–14 days until significant cytopathic effects were observed. Supernatants were then collected, aliquoted, and frozen at −80°C as “passage 1” virus stocks. The amount of infectious virus in the stocks was determined by a FFU assay, with titers ranging from 10^6^ to 10^7^ FFU/mL. To confirm that no mutations arose during virus rescue, we isolated viral RNA from each passage 1 virus stock, converted it to cDNA, and sequenced a portion of each genome. No mutations were observed in any of the passage 1 virus stocks, which all matched the GenBank records perfectly (Tables S3).

**TABLE 1:** Table of sylvatic dengue virus sequences available on GenBank (in bold are stocks rescued here)^*f*^

Type	Strain	Year isolated	Origin	Host isolated from	GenBank accession number	BEI catalog number^*g*^
1	**Brun2014** ^***a***^	2014	Brunei^*b*^	*Homo sapiens*	KR919820	-
	**P72-1244**	1972	Malaysia	Sentinel monkey	EF457905	-
2	Dak Ar A2022^*c*^	1980	Burkina Faso	*Aedes africanus*	EF105386	-
	PM33974^*c*^	1981	Guinea	*Aedes africanus*	EF105378	NR-12222
	Dak Ar D20761^*c*^	1974	Senegal	*Aedes luteocephalus*	EF105385	-
	Dak Ar 2039^*c*^	1980	Burkina Faso	*Aedes luteocephalus*	EF105382	-
	Dak Ar D75505^*c*^	1999	Senegal	*Aedes luteocephalus*	EF457904	-
	Dak Ar 141069^*c*^	1999	Senegal	*Aedes luteocephalus*	EF105389	-
	Dak Ar 141070^*c*^	1999	Senegal	*Aedes luteocephalus*	EF105390	-
	**Dak Ar A1247** ^***c***^	1980	Cote d'Ivoire	*Aedes taylori*	EF105383	NR-12221
	Dak Ar 578^*c*^	1980	Cote d'Ivoire	*Aedes taylori*	EF105380	-
	Dak Ar 510^*c*^	1980	Cote d'Ivoire	*Aedes taylori*	EF105381	-
	Dak HD 76395^*c*^	1990	Senegal	*Homo sapiens*	OK605757	-
	**DKD811** ^***a***^	2008	Malaysia	*Homo sapiens*	FJ467493	NR-49747^*d*^
	EEB-17^*a*^^,*e*^	2009	Guinea-Bissau	*Homo sapiens*	JF260983	-
	**DSab2015/QML22** ^***a***^	2015	Malaysia^*b*^	*Homo sapiens*	KY923048/KX274130	-
	IBH11234^*c*,*e*^	1966	Nigeria	*Homo sapiens*	EU003591	-
	IBH11208^*c*^	1966	Nigeria	*Homo sapiens*	EF105387	-
	**IBH11664** ^***c***^	1966	Nigeria	*Homo sapiens*	EF105388	-
	Dak HD 10674^*c*^	1970	Senegal	*Homo sapiens,*	EF105384	-
	P8-1407^*c*^	1970	Malaysia	Sentinel monkey	EF105379	NR-3790
	SH356683^*a*,*e*^	2020	Senegal	*Homo sapiens*	PP029070	-
	SH356692^*a*,*e*^	2020	Senegal	*Homo sapiens*	PP029068	-
	SH356702^*a*,*e*^	2020	Senegal	*Homo sapiens*	PP029069	-
4	P75-215	1975	Malaysia	*Aedes niveus s. l*.	EF457906	-
	DKE-121	2007	Malaysia	*Homo sapiens*	MZ215848	-
	**P73-1120** ^***c***^	1973	Malaysia	Sentinel monkey	JF262780	-
	P75-514^*c*^	1975	Malaysia	Silver Leaf Monkey	JF262779	-

^*a*^ Sequences for these strains were generated from virus isolates obtained from human patient samples.

^*b*^ These viruses were isolated in Australia from travelers returning from the origin listed.

^*c*^ Sequences for these strains were generated from virus isolates at the UTMB World Reference Center for Emerging Viruses and Arboviruses and from the Institut Pasteur de Dakar, Dakar, Senegal.

^*d*^ A variant of DKD811 that shares >99% sequence identity to DKD811 is available on BEI Resources.

^*e*^ Sequences for these strains are missing parts of the untranslated region.

^*f*^ Strains that are bolded were rescued in this study.

^*g*^ - indicates not available.

### Only some sylvatic dengue virus strains replicate in human cells

**Fig 5:**
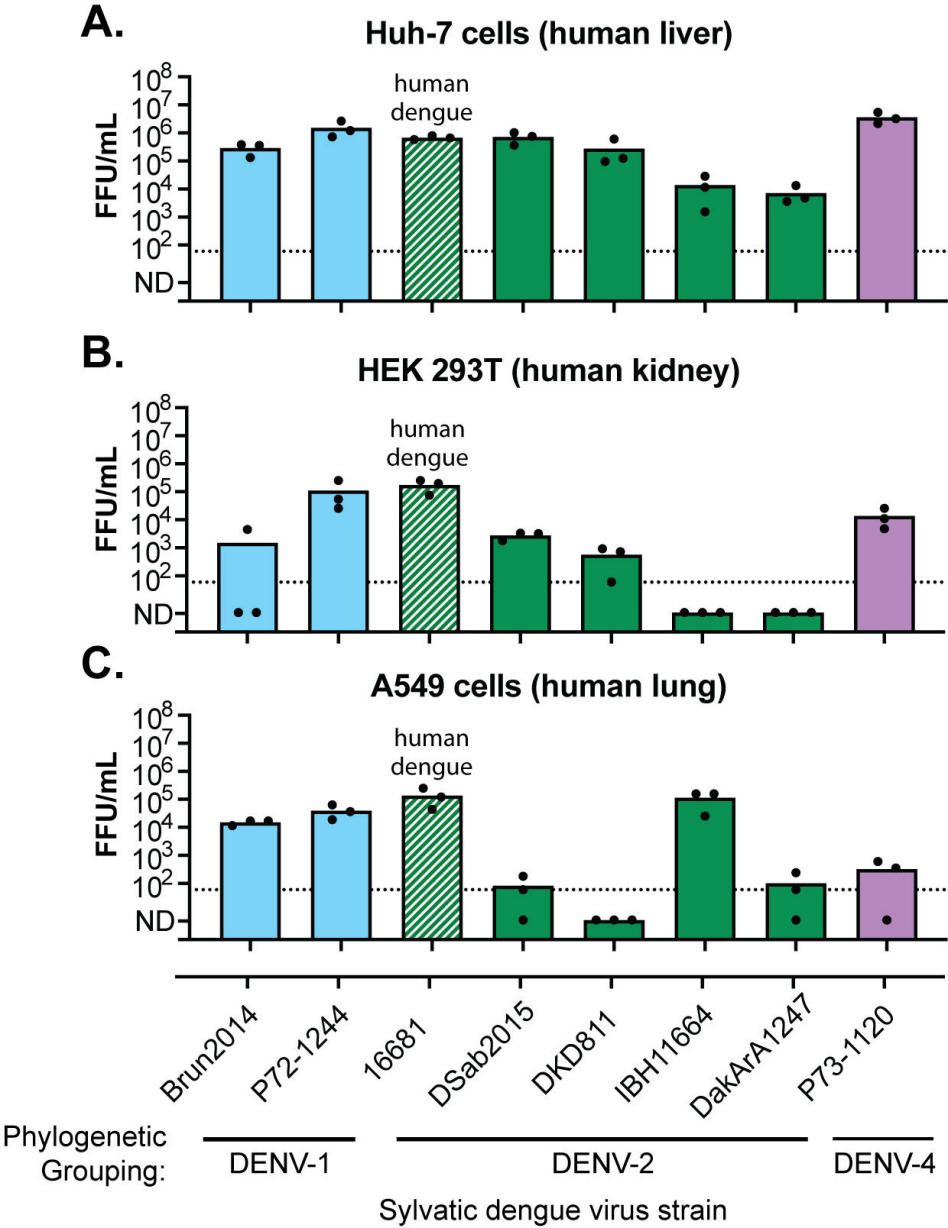
Only some sylvatic dengue virus strains replicate in human cells. (A–C) The indicated human cell lines (top of graphs) were exposed to eight launched stocks of dengue virus (virus strains denoted on X axis) at a multiplicity of infection of 0.1. All are sylvatic strains except 16681, which is a human dengue virus control. Supernatants were collected 3 days post-exposure, and virus titers were determined by FFU assay (Y axis). Each dot represents a technical replicate, with the top of the bar representing the mean of all three technical replicates. The dotted line represents the limit of detection of the assay. Values under the limit of detection were assigned a value of 1 FFU/mL for the purpose of calculating the mean and are plotted at “not-detected” (N.D.) on the Y axis.

Now possessing this unique collection of sylvatic dengue viruses, we exposed a panel of human cells to these viruses. The goal was to determine if some sylvatic dengue viruses are more “pre-adapted” for replication in human cells. Three human cell lines were each exposed to one of the seven launched stocks of sylvatic dengue virus, as well as the launched stock of a human dengue virus (strain 16681, launched in Fig. 3), at a multiplicity of infection of 0.1. The titer of each virus, obtained after 3 days of replication on each cell line, is plotted (Fig. 5). Several observations can be made. First, the human dengue virus strain replicated robustly on all three cell lines, as expected, since these cell lines are commonly used to propagate human dengue viruses in the literature. Second, only some sylvatic dengue viruses replicated high titers in human cell lines. In general, sylvatic dengue viruses replicated best on Huh-7 (liver) cells. However, only some strains of sylvatic dengue viruses were able to replicate on HEK 293T (kidney) and A549 (lung) cell lines. This could mean that these cell lines contain restriction factors that act against sylvatic (but not human) dengue viruses, or these cells do not express certain human proteins that sylvatic (but not human) dengue viruses need to hijack from the cell to replicate. While these data are preliminary, they are the first to show biological differences between sylvatic dengue virus strains, and that some sylvatic viruses replicate better than others on human cells.

### Only some sylvatic dengue virus strains replicate in African green monkey cells

**Fig 6:**
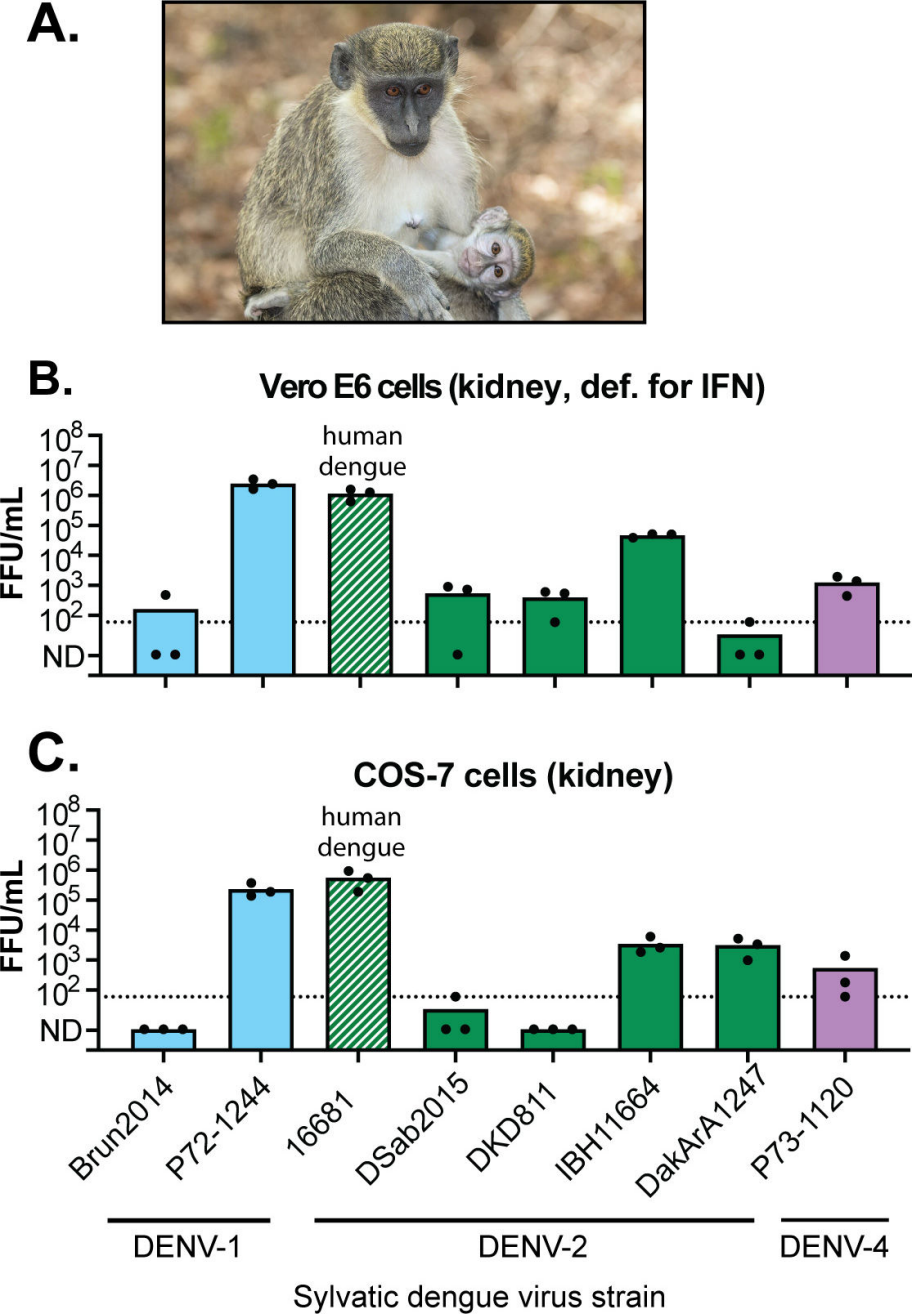
Only some sylvatic dengue virus strains replicate in monkey cells. (A) African green monkey, *Chlorocebus sabaeus*, photographed in The Gambia by Charles Sharp and used under CC BY-SA 4.0. (B and C) The indicated cell lines (top of graphs) were exposed to one of eight launched stocks of dengue virus (virus strains denoted on X axis) at a multiplicity of infection of 0.1. All are sylvatic strains except 16681, which is a human dengue virus control. Supernatants were collected 3 days post-exposure, and virus titers were determined by FFU assay (Y axis). Each dot represents a technical replicate, with the top of the bar representing the mean of all three technical replicates. The dotted line represents the limit of detection of the assay. Values under the limit of detection were assigned a value of 1 FFU/mL for the purpose of calculating the mean and are plotted at “not-detected” (N.D.) on the Y axis.

Next, we performed the same replication assay in two African green monkey cell lines. African green monkeys (*Chlorocebus sabaeus*; Fig. 6A) are a suspected host of sylvatic dengue viruses in their natural reservoir in West Africa (13). The human dengue virus strain 16681 replicated robustly on both cell lines, generating 10^6^ infectious virions per milliliter in the supernatant over 3 days. Only sylvatic dengue virus strain P72-1244 grew to similar titers in African green monkey cells (Fig. 6B and C). Interestingly, this is one of the few sylvatic viruses ever sequenced that was isolated outside of Africa (Table 1). This strain was sampled from a sentinel monkey (most likely *Macaca fascicularis* [crab-eating macaque] or *Presbytis obscura* [dusky leaf monkey]) in Malaysia (14, 20, 63). The sylvatic strains IBH11664, DakArA1247, and P73-1120 were intermediate at replicating on these monkey cells, while other sylvatic virus strains did not replicate well. African green monkeys have been experimentally exposed *in vivo* to one sylvatic dengue virus strain (not included in our panel), and the virus replicated poorly (26). Our data could indicate that different results might be obtained with different sylvatic virus strains. Finally, it should be noted that while the two cell lines used here are both derived from African green monkey kidneys, the Vero E6 line does not express type I interferon, and other aspects of the interferon response in this cell line are hypomorphic as well (57–59). We note that there is not a profound difference in the ability of human or sylvatic viruses tested here to replicate on one cell line versus the other, suggesting that the viruses tested here are capable of circumventing at least some aspects of the interferon response pathway and are thus not sensitive to its integrity.

### All tested sylvatic dengue virus strains replicate in mosquito cells

**Fig 7:**
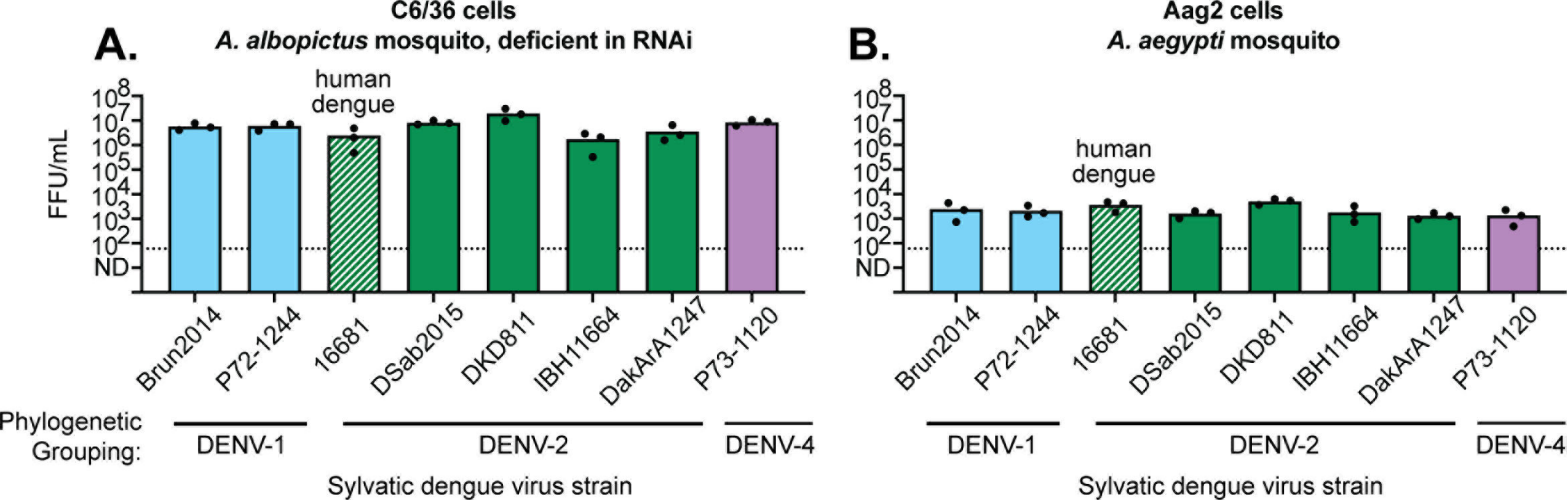
All tested sylvatic dengue virus strains replicate in mosquito cells. (A, B) The indicated cell lines (top of graphs) were exposed to one of eight launched stocks of dengue virus (X axis) at a multiplicity of infection of 0.1. All are sylvatic strains except 16681, which is a human dengue virus control. Supernatants were collected 3 days post-exposure, and virus titers were determined by FFU assay. The Y-axis represents FFUs per milliliter. Each dot represents a technical replicate, with the top of the bar representing the mean of all three technical replicates. The dotted line represents the limit of detection of the assay.

Finally, we exposed two mosquito cell lines—the *Aedes albopictus* C6/36 cell line and the *Aedes aegypti* Aag2 cell line—to our panel of rescued dengue viruses. The concentration of infectious virus in the supernatant, achieved after 3 days of replication on each cell line, is plotted (Fig. 7). All viruses replicated to consistent levels in mosquito cells, unlike the variation observed in human and monkey cell lines. This supports the idea that C6/36 cells are more universally permissive for the replication of sylvatic dengue viruses than are mammalian cell lines. Interestingly, C6/36 cells generated far more virus than Aag2 cells, likely due to their deficiency in RNAi signaling (62).

## DISCUSSION

Over the last 20 years, sylvatic dengue viruses have spilled over into humans at least eight times and even caused a small-scale epidemic in Senegal (Fig. 1). Additionally, sylvatic dengue viruses have caused four human pandemics (DENV-1, 2, 3, and 4), though the details are lost to history. Despite their potential threat, these viruses remain significantly understudied, largely due to the limited availability of infectious stocks. Reverse genetics offers a solution to this problem, allowing researchers to transform genome sequences into infectious viral stocks. In our search for a broadly permissive cell line to recover dengue virus strains, we found that *Aedes albopictus* C6/36 and *Aedes aegypti* Aag2 cell lines supported the launch of seven diverse sylvatic dengue virus strains. This was not surprising, as *Aedes albopictus* and *Aedes aegypti* are the primary vectors of human dengue viruses, and sylvatic dengue viruses have been isolated from multiple *Aedes* species (Table 1). The *Aedes albopictus* C6/36 cell line exhibited the highest efficiency for both launch and subsequent virus propagation, making it an ideal candidate for recovering a wide range of dengue viruses, including sylvatic strains. This broad permissiveness is likely due to the absence of a functional RNAi pathway in C6/36 cells, which otherwise acts as a key antiviral defense against orthoflaviviruses in mosquitoes.

Using this system, we generated seven infectious stocks, enabling us to characterize these previously unstudied or understudied sylvatic dengue virus strains and gain insights into their biology. We find that sylvatic dengue viruses exhibit striking variability in their ability to infect mammalian host cells. While a human dengue virus replicated efficiently in all tested human cell types, only certain sylvatic strains showed robust replication, suggesting that host restriction factors may limit their ability to infect humans. Similarly, replication in African green monkey cells varied widely, with some sylvatic strains failing to replicate even in Vero E6 cells, which lack interferon signaling. This suggests that additional, unidentified host factors may contribute to viral restriction. Importantly, some sylvatic dengue viruses appear to have evolved mechanisms to overcome these barriers, while others remain restricted. Understanding these differences provides a framework for investigating how sylvatic dengue viruses adapt to new hosts and may help identify viral determinants that drive host adaptation. For instance, we have recently shown that dengue viruses cleave the human but not nonhuman primate STING protein, which acts in innate immunity (64). Further work will be needed to determine if some or all sylvatic dengue viruses have this property.

Overall, our study highlights the utility of mosquito cells as a broadly permissive platform for rescuing diverse sylvatic dengue virus strains and demonstrates the power of reverse genetics in expanding access to previously inaccessible viruses. This, together with new tools for propagating more biologically relevant dengue target cells from human and primate stem cells (65), lays the foundation for further characterization of unstudied and diverse sylvatic dengue viruses, ultimately advancing our understanding of their biology, host-virus interactions, and spillover potential.

## MATERIALS AND METHODS

### Cells and viruses

Human kidney (HEK 293T, ATCC, Cat#11268), human lung carcinoma (A549, ATCC, Cat#CCL-185), human hepatoma (Huh7, JCRB, Cat#0403), and African green monkey kidney (COS-7, ATCC, Cat#CRL-1651) cells were maintained in Dulbecco’s Modified Eagle’s Medium (DMEM, MilliporeSigma, #D6429) supplemented with 10% fetal bovine serum (FBS, MilliporeSigma, TMS-013-B) and 1% L-glutamine (Corning Incorporated, #MT25002CI). Grivet monkey kidney Vero E6 (Vero, ATCC, Cat#CRL-1586), baby hamster kidney (BHK-21, ATCC, Cat#CCL-10), *Aedes albopictus* mosquito (C6/36, ATCC, Cat#CRL-1660), and *Aedes aegypti* mosquito (Aag2) cells were maintained in Eagle’s Minimum Essential Medium (EMEM, ATCC, 30–2003) supplemented with 10% FBS and 1% L-glutamine. All mammalian (human, monkey, and hamster) cells were grown at 37°C, while all mosquito cells were maintained at 28°C. All cells were incubated in 5% CO_2_. For transfection with CPER products and for virus exposures, heat-inactivated FBS was used and lowered to 2%–5%. Cells were seeded at the following densities per well in 12-well plates: 425k 293T, 150k Vero E6, 200k BHK, 200K A549, 100k COS-7, 400k Huh-7, 425k C6/36, and 300k Aag2. For Aag2 cells, 400 µL of poly-L-lysine (Sigma-Aldrich, Cat#P4707) was used to coat each well prior to seeding.

Sylvatic DakArA1247 was obtained from BEI Resources (Cat#NR-12221). All other sylvatic dengue viruses were rescued in this study, based on the following GenBank accession numbers: Brun2014 (KR919820), P72-1244 (EF457905), DakArA1247 (EF105383), DKD811 (FJ467493), DSab2015 (KY923048), IBH11664 (EF105388), and P73-1120 (JF262780).

### Circular polymerase extension reaction

Dengue genomes were synthesized as overlapping fragments by Twist Biosciences. Typically, fragments were ordered as follows: fragment 1 (bases 1–3,000), fragment 2 (bases 2,800–7,000), and fragment 3 (6,800–10,700/end of the genome). Each fragment was designed with ~200 base pair overlaps with adjacent fragments to facilitate primer design for CPER assembly.

Occasionally, synthesis challenges were encountered due to the dengue virus’ genomic toxicity or instability in bacteria, necessitating adjustments in fragment design. For example, for P72-1244, fragment 1 was split into three smaller overlapping fragments (fragments 1A: bases 1–688, 1B: bases 636–2,226, and 1C: bases 2,190–3,549), and fragment 3 was split into two smaller overlapping fragments (fragments 3A: bases 7,100–9,722 and 3B: bases 9,696–10,735). These smaller fragments were assembled using overlap PCR (66), where overlapping regions served as primers for adjacent fragments, facilitating polymerase-mediated joining, similar to how the circular polymerase extension reaction works. Once assembled, the original fragments 1–3 were PCR amplified from the overlap PCR products and sequenced prior to performing CPER.

We demonstrate that another option to sidestep potential DNA synthesis problems is to order smaller overlapping fragments from the start. For example, seven fragments spanning the DSab2015 genome were ordered: fragment 1 (bases 1–1,019); fragment 2 (bases 920–2,719); fragment 3 (bases 2,620–4,419); fragment 4 (bases 4,320–6,119); fragment 5 (bases 6,020–7,819); fragment 6 (bases 7,720–9,519); and fragment 7 (bases 9,420–10,737). These fragments, along with a UTR-linker, were used to perform CPER.

The circular polymerase extension reaction was then performed by amplifying overlapping PCR fragments of approximately 1.0–4.3 kb in length. The overlap regions between adjacent fragments were generated to be 18–20 bases long, with a melting temperature (Tm) of approximately 63°C, calculated using the NEB Tm Calculator for Q5 2× Master Mix (tmcalculator.neb.com). For example, DakArA1247 fragment 1 (bases 1–2,836) and fragment 2 (bases 2,819–6,954) were amplified with primers that produced an 18-nucleotide overlap (bases 2,819–2,836), with a Tm of 63°C, verified by the NEB Tm Calculator. The start of fragment 1 and the end of fragment 3 were also designed to share overlaps with the UTR linker. All primers used to generate PCR fragments for the circular polymerase extension reaction can be found in Tables S1 and S2.

The UTR-linkers were designed to include the hepatitis delta virus ribozyme/simian 40 polyadenylation signal (HDR/SV40pA) sequence, a linker region, and either a cytomegalovirus promoter, as used in Conde et al., or an OpIE2 promoter (52). Sequences for both UTR linkers were obtained from Dr. Jonas Nascimento Conde and synthesized by commercial services. Detailed sequences of both UTR linkers are provided in Fig. S1.

PCR products were purified using the Wizard SV Gel and PCR Clean-Up System (Promega, A9282) following the manufacturer’s “DNA Purification by Centrifugation” Quick Protocol. Purified fragments were combined with Q5 High-Fidelity 2× Master Mix (New England Biolabs, Cat#M0492S) at a ratio of 0.1 pmol/fragment and subjected to the following thermocycling conditions: 98°C for 30 seconds, followed by 12 cycles of 98°C for 10 seconds, 60°C for 20 seconds, and 72°C for 10 min, with a final extension at 72°C step for 10 min. The resulting CPER products were either stored at −20°C or directly transfected into cells.

### Launch of infectious virus from CPER-assembled genomes

All cells were transfected using TransIT-LT1 (Mirus Bio, MIR 2300). TransIT-Insect (Mirus Bio, MIR 6100) was initially used for transfecting mosquito cells but was found to be more toxic than TransIT-LT1. For each transfection, 20 µL of the circular polymerase extension reaction was mixed with 100 µL of Opti-MEM Reduced Serum Medium (Thermo Fisher Scientific, 31985–062) and 3 µL of TransIT-LT1. This mixture was incubated at room temperature for 10–15 min and then added dropwise directly onto cells. Cells were seeded to 80% confluence in either DMEM or EMEM, supplemented with 10% FBS and 1% L-glutamine. Prior to transfection, media was replaced to contain 3%–5% heat-inactivated FBS.

C6/36 cells were transfected with sylvatic dengue virus genomes assembled by the circular polymerase extension reaction with the linker containing the insect OpIE2 promoter. Seven days post-transfection, 500 µL of C6/36 cell supernatant was collected, which contained passage 0 (P0) virus. The supernatant was directly added to a 15 cm plate of C6/36 cells, and the virus was allowed to propagate for 7–14 days until significant CPE was observed. Upon detection of CPE, cell supernatants (now containing passage 1 [P1] virus) were collected, aliquoted, and stored at −80°C. Stocks were then analyzed to determine the concentration of infectious virus per milliliter using a focus-forming unit assay.

### Focus-forming unit assay for enumerating infectious virus titers

A focus-forming unit assay was performed to determine virus titers. C6/36 cells were seeded at 250,000 cells/well in a 24-well plate, in EMEM supplemented with 10% FBS and 1% L-glutamine. After 24 h, viral dilutions were prepared in Dulbecco’s phosphate-buffered saline (DPBS) containing calcium and magnesium (Corning 21–030-CM). Media was removed from the cells, and 165 µL of virus dilution was added per well for 1 h at 28°C, with gentle rocking every 15 min. After the incubation, viral dilutions were aspirated, and 1 mL of overlay media was added to each well. The overlay consisted of 1.2× Avicel (FMC Corporation, RC-591 NF), 2% heat-inactivated FBS, 1× non-essential amino acids, 1× L-glutamine, and 1× EMEM. Cells were then incubated at 28°C for 4 days.

Four days post-exposure, 1 mL of PBS was added to each well, and the overlay media was aspirated. The cells were washed once with PBS and then fixed in 400 µL of 4% paraformaldehyde (PFA) for 15 min. After removing the PFA, cells were air-dried for 10 min at room temperature. Cells were then washed with blocking buffer (PBS + 3% FBS) three times. To detect viral foci, cells were incubated with dengue virus type two strain New Guinea C (NGC) hyperimmune mouse ascitic fluid (BEI Resources Cat#NR-51426) diluted 1:1,000 in a blocking buffer for 30 min. After washing the cells three more times with blocking buffer, a rabbit anti-mouse IgG (H + L) secondary antibody-HRP stain (Invitrogen, Cat#PA1-28568) was added at 1:1,000 in a blocking buffer for 30 min. The cells were washed three more times and then incubated with aminoethylcarbazole (AEC) peroxidase substrate (Enzo Life Sciences, ENZ-43825) for 15 min. The cells were gently washed with water and left to air dry. Foci were counted over a light box.

### Virus exposures

For viral exposures, cells were seeded in either DMEM or EMEM, both supplemented with 10% FBS and 1% L-glutamine, at the following densities per well in 24-well plates: 200k HEK 293T, 65k Vero E6, 100K A549, 65k COS-7, 200k Huh7, 250k C6/36, and 150k Aag2. Prior to seeding Aag2 cells, 200 µL of poly-L-lysine (Sigma-Aldrich, Cat#P4707) was used to coat each well. After 24 h, viral dilutions were made in DPBS containing calcium and magnesium (Corning 21–030-CM) to the desired concentrations (e.g., multiplicity of infection of 0.1). The media was aspirated off the cells, and viral dilutions were added. Cells were then incubated at 37°C for mammalian cells or at 28°C for mosquito cells for 1 h, with gentle rocking every 15 min. After the incubation, the viral solution was aspirated, and cells were washed twice with DPBS containing calcium and magnesium. One milliliter of infection media was then added to each well.

For C6/36 cells, the exposure infection media consisted of EMEM with 2% heat-inactivated FBS, 1× non-essential amino acids (HyClone SH30238.01), 1× L-glutamine, and 25 mM HEPES buffer (HyClone SH30237.01)). For Aag2 cells, Schnider’s insect media (Sigma-Aldrich, Cat#S0146) was used with 2% heat-inactivated FBS, 1× non-essential amino acids (HyClone SH30238.01), 1× L-glutamine, and Amphotericin B (Sigma-Aldrich, Cat#A2942). For mammalian cells, DMEM or EMEM was supplemented with 5% heat-inactivated FBS and 1× L-glutamine. Cells were incubated at 37°C for mammalian cells or 28°C for mosquito cells.

### vRNA extractions

Viral RNA was isolated using the QIAamp Viral RNA Mini Kit (Qiagen, 52904) following the “Purification of Viral RNA (Spin Protocol)” from the QIAamp Viral RNA Mini Handbook 07/2020. Briefly, 140 µL of supernatant was collected and mixed with viral lysis buffer (AVL buffer) and then frozen at −80°C. After thawing, 100% ethanol was added to the AVL buffer, and the mixture was passed through the affinity column. RNA was washed twice with the provided wash buffers and then eluted with water. The purified RNA was then aliquoted and stored at −80°C until further use.

### RT-qPCR for enumeration of total virions

RT-qPCR was performed on isolated viral RNA using the NEB Luna Universal One-Step RT-qPCR kit (Cat#E3005), following the manufacturer’s protocol. Serotype-specific dengue primers were used for amplification (primer sequences from https://journals.asm.org/doi/10.1128/jcm.43.10.4977-4983.2005). Reactions were run on a QuantStudio3 PCR machine under the following conditions: reverse transcription at 55°C for 10 min, initial denaturation at 95°C for 1 min, followed by 45 cycles of denaturation at 95°C for 10 seconds, and extension at 60°C for 30 seconds with the plate reading at the end of each cycle. A melt curve from 60-95°C was performed at the end of the amplification. Standards for dengue type 2 were prepared using synthetic dengue virus type 2 RNA (ATCC VR-3229SD).

### cDNA synthesis and sequencing

cDNA was synthesized from extracted viral RNA using the SuperScript IV First-Strand Synthesis System (Invitrogen, Cat#18091050), following the manufacturer’s guidelines (Pub. No. MAN0013442). PCR amplification of the cDNA was performed using appropriate primers for the target sequences. The resulting PCR amplicons were purified using the Wizard SV Gel and PCR Clean-Up System (Promega, Cat#A9282). Purified amplicons were then sent for sequencing by Oxford Nanopore Technologies via Plasmidsaurus.

## Data Availability

All data are available within this paper and its supplemental material.

## SUPPLEMENTAL MATERIAL

Supplemental tables and figures jvi.00450-25-s0001.docx Tables S1 to S3 and Fig. S1 to S2.

ASM does not own the copyrights to Supplemental Material that may be linked to, or accessed through, an article. The authors have granted ASM a non-exclusive, world-wide license to publish the Supplemental Material files. Please contact the corresponding author directly for reuse.
