# Supplementary material for: Reverse genetics rescue of sylvatic dengue viruses
Source: J Virol. 2025 Jun 4;99(7):e00450-25. doi: 10.1128/jvi.00450-25 (PMC12282064; doi:10.1128/jvi.00450-25)
Supplement: Supplemental tables and figures — Tables S1 to S3 and Fig. S1 to S2. [file jvi.00450-25-s0001.docx]

**Supplementary Information**

| Virus | Fragment | Length | Primer | Primer sequence |
| --- | --- | --- | --- | --- |
| 16681 | F1, CMV | 2913 | 16681.CMV.1F | GTTTAGTGAACCG**AGTTGTTAGTCTACGTGG** |
|  |  |  | 16681.1R | GGTGAATACTCCAAAGCC |
|  | F1, OpIE2 | 2913 | 16681.OpIE2.1F | CGATCTGGTAAAC**AGTTGTTAGTCTACGTGG** |
|  |  |  | 16681.1R | GGTGAATACTCCAAAGCC |
|  | F2 | 4142 | 16681.2F | TGGCTTTGGAGTATTCACC |
|  |  |  | 16681.2R | GGTTGGCTATAGCTGTTAG |
|  | F3 | 3706 | 16681.3F | CCTAACAGCTATAGCCAAC |
|  |  |  | 16681.3R | GCCGACCC**AGAACCTG** |
| DakAr1247 | F1, CMV | 2805 | Dak.CMV.1F | GTTTAGTGAACCG**AGTTGTTAGTCTACGTGGAC** |
|  |  |  | Dak.1R | CAGGACCGTCTATGAGG |
|  | F1, OpIE2 | 2805 | Dak.OpIE2.1F | CGATCTGGTAAAC**AGTTGTTAGTCTACGTGGAC** |
|  |  |  | Dak.1R | CAGGACCGTCTATGAGG |
|  | F2 | 4100 | Dak.2F | TCCTCATAGACGGTCC |
|  |  |  | Dak.2R | CACTGCATATAGTGTCCAC |
|  | F3 | 3736 | Dak.3F | CGTGGACACTATATGCAG |
|  |  |  | Dak.3R | GCCGACCC**AGAACCTGTTGATTCAACAG** |
| P72-1244 | F1, OpIE2 | 3419 | P72.1F | CGATCTGGTAAAC**AGTTGTTAGTCTACGTGGAC** |
|  |  |  | P72.1R | CCATGCCATACCAACATC |
|  | F2 | 4182 | P72.2F | GGATGTTGGTATGGCATGG |
|  |  |  | P72.2R | GCATAATGAGCCACTAGCATC |
|  | F3 | 3576 | P72.3F | GTGTTGATGCTAGTGGCTCATTATG |
|  |  |  | P72.3R | GCCGACCC**AGAACCTGTTGATTCAACAG** |
| P73-1120 | F1, CMV | 2801 | P73.CMV.1F | GTTTAGTGAACCG**AGTTGTTAGTCTGT** |
|  |  |  | P73.1R | GGTCCATCTATCAGGAATG |
|  | F1, OpIE2 | 2801 | P73.OpIE2.1F | CGATCTGGTAAAC**AGTTGTTAGTCTGT** |
|  |  |  | P73.1R | GGTCCATCTATCAGGAATG |
|  | F2 | 4140 | P73.2F | CACATTCCTGATAGATGGACC |
|  |  |  | P73.2R | GTAGTTGCTACTGCATAGAG |
|  | F3 | 3734 | P73.3F | CACTCTATGCAGTAGCAACTAC |
|  |  |  | P73.3R | GCCGACCC**AGAACCTGTTGGATCAAC** |
| Brun2014 | F1, CMV | 2797 | Brun.CMV.1F | GTTTAGTGAACCG**AGTTGTTAGTCTACGTGG** |
|  |  |  | Brun.1R | GCAGCAGACATCAGTC |
|  | F1, OpIE2 | 2797 | Brun.OpIE2.1F | CGATCTGGTAAAC**AGTTGTTAGTCTACGTGG** |
|  |  |  | Brun.1R | GCAGCAGACATCAGTC |

**Supplementary Table 1 (continued)**

| Virus | Fragment | Length | Primer | Primer sequence |
| --- | --- | --- | --- | --- |
| Brun2014 | F2 | 4047 | Brun.2F | CAGACTGATGTCTGCTGC |
|  |  |  | Brun.2R | GTTGTGTTCTCTATTGTGTGC |
|  | F3 | 3737 | Brun.3F | GCACACAATAGAGAACACAAC |
|  |  |  | Brun.3R | GCCGACCC**AGAACCTGTTGATTCAACAG** |
| IBH16644 | F1, OpIE2 | 2838 | IBH.1F | CGATCTGGTAAAC**AGTTGTTAGTCTACGTGG** |
|  |  |  | IBH.1R | CAGGACCATCTATGAGG |
|  | F2 | 4128 | IBH.2F | CTTTCCTCATAGATGGTCC |
|  |  |  | IBH.2R | GTGTCCACGCTGATG |
|  | F3 | 3784 | IBH.3F | GCATCAGCGTGGACAC |
|  |  |  | IBH.3R | GCCGACCC**AGAACCTGTTGATTCAACAGCAC** |
| DKD811 | F1, OpIE2 | 2867 | DKD.1F | CGATCTGGTAAAC**AGTTGTTAGTCTACGTGG** |
|  |  |  | DKD.1R | GCCCTGTTGCTGTTAG |
|  | F2 | 4098 | DKD.2F | CCTAACAGCAACAGGG |
|  |  |  | DKD.2R | GTATAGTGTCCATGCTGATG |
|  | F3 | 3792 | DKD.3F | GCATCAGCATGGACACTATAC |
|  |  |  | DKD.3R | GCCGACCC**AGAACCTGTTGATTCAACAGCAC** |
| DSab2015 | F1, OpIE2 | 1018 | DSab.1F | CGATCTGGTAAAC**AGTTGTTAGTCTACGTGG** |
|  |  |  | DSab.1R | CCATGTTCCAAGACAATGTC |
|  | F2 | 1659 | DSab.2F | TTGACATTGTCTTGGAACATG |
|  |  |  | DSab.2R | GGATGTGATTTAGTTCTGGG |
|  | F3 | 1744 | DSab.3F | CCCCAGAACTAAATCACATC |
|  |  |  | DSab.3R | CTACCATCTTCTGATACAGCC |
|  | F4 | 1719 | DSab.4F | TGGCTGTATCAGAAGATGG |
|  |  |  | DSab.4R | TGGCATCAGTTTTTTCCC |
|  | F5 | 1713 | DSab.5F | GAGGGAAAAAACTGATGCC |
|  |  |  | DSab.5R | TCCACGAACCATCTCAG |
|  | F6 | 1691 | DSab.6F | ACTGAGATGGTTCGTGG |
|  |  |  | DSab.6R | CCCCTTCCATTTGCC |
|  | F7 | 1315 | DSab.7F | CATTAGGCAAATGGAAGGG |
|  |  |  | DSab.7R | GCCGACCC**AGAACCTGTTGATTCAACAG** |

**Table S1: List of PCR primers for generation of fragments used as input to the circular polymerase extension reactions.** Each fragment for was generated by PCR with the primers above. Certain primers contained overhang sequences complementary to a UTR linker. UTR linker sequences are underlined, while dengue genomic sequences are bolded.

| Linker | Primer type | Length | Primer name | Primer sequence |
| --- | --- | --- | --- | --- |
| OpIE2 UTR Linker | Universal | 958 | OpIE2.1F | **AACAGGTTCT**GGGTCGGCATGGCATCTC |
|  |  |  | OpIE2.1R | **GTAGACTAACAACT**GTTTACCAGATCGTTGCGGGC |
|  | P73-1120 | 958 | OpIE2.2F | **AACAGGTTCT**GGGTCGGCATGGCATCTC |
|  |  |  | OpIE2.2R | **ACAGACTAACAACT**GTTTACCAGATCGTTGCGGGC |
| CMV UTR Linker | Universal | 782 | CMV.1F | **CTGTTGAATCAACAGGTTCT**GGGTCGGCATGGCATCTC |
|  |  |  | CMV.1R | **CCACGTAGACTAACAACT**CGGTTCACTAAACGAGC |
|  | P73-1120 | 782 | CMV.2F | **TTGTTGATCCAACAGGTTCT**GGGTCGGCATGGCATCTC |
|  |  |  | CMV.2R | **CCACACAGACTAACAACT**CGGTTCACTAAACGAGC |

**Table S2: List of primers used to generate different versions of the UTR-linker fragment.** Each UTR linker fragment was generated by PCR with the primers above. Because the P73-1120 sequence had a couple nucleotide differences in the first 18 bases of the genome, P73-1120 specific primers were designed to amplify a P73-1120 specific UTR linker. Universal primers were used to generate UTR linkers that were compatible with all remaining strains of virus rescued. UTR linker sequences are underlined, while dengue genomic sequences are bolded.

**OpIE2-UTR-Linker**

GGGTCGGCATGGCATCTCCACCTCCTCGCGGTCCGACCTGGGCATCCGAAGGAGGACGTCGTCCACTCGGATGGCTAAGGGAGAGCCACTTTTCTCTCGATTCTCTATCGGAATCTAGGGAGCTCGGATCCAGACATGATAAGATACATTGATGAGTTTGGACAAACCACAACTAGAATGCAGTGAAAAAAATGCTTTATTTGTGAAATTTGTGATGCTATTGCTTTATTTGTAACCATTATAAGCTGCAATAAACAAGTTAACAACAACAATTGCTCGAGGGGGGGCCCGGTACCTTGAAGCTGTCCCTGATGGTCGTCATCTACCTGCCTGGACAGCATGGCCTGCAACGCGGGCATCCCGATGCCGCCGGAAGCGAGAAGAATCATAATGGGGAAGGCCATCCAGCCTCGCGTCGGCGCTTAAGGATCATGATGATAAACAATGTATGGTGCTAATGTTGCTTCAACAACAATTCTGTTGAACTGTGTTTTCATGTTTGCCAACAAGCACCTTTATACTCGGTGGCCTCCCCACCACCAACTTTTTTGCACTGCAAAAAAACACGCTTTTGCACGCGGGCCCATACATAGTACAAACTCTACGTTTCGTAGACTATTTTACATAAATAGTCTACACCGTTGTATACGCTCCAAATACACTACCACACATTGAACCTTTTTGCAGTGCAAAAAAGTACGTGTCGGCAGTCACGTAGGCCGGCCTTATCGGGTCGCGTCCTGTCACGTACGAATCACATTATCGGACCGGACGAGTGTTGTCTTATCGTGACAGGACGCCAGCTTCCTGTGTTGCTAACCGCAGCCGGACGCAACTCCTTATCGGAACAGGACGCGCCTCCATATCAGCCGCGCGTTATCTCATGCGCGTGACCGGACACGAGGCGCCCGTCCCGCTTATCGCGCCTATAAATACAGCCCGCAACGATCTGGTAAAC

**CMV-UTR-Linker**

GGGTCGGCATGGCATCTCCACCTCCTCGCGGTCCGACCTGGGCATCCGAAGGAGGACGTCGTCCACTCGGATGGCTAAGGGAGAGCCACTTTTCTCTCGATTCTCTATCGGAATCTAGGGAGCTCGGATCCAGACATGATAAGATACATTGATGAGTTTGGACAAACCACAACTAGAATGCAGTGAAAAAAATGCTTTATTTGTGAAATTTGTGATGCTATTGCTTTATTTGTAACCATTATAAGCTGCAATAAACAAGTTGTTACATAACTTACGGTAAATGGCCCGCCTGGCTGACCGCCCAACGACCCCCGCCCATTGACGTCAATAATGACGTATGTTCCCATAGTAACGCCAATAGGGACTTTCCATTGACGTCAATGGGTGGAGTATTTACGGTAAACTGCCCACTTGGCAGTACATCAAGTGTATCATATGCCAAGTACGCCCCCTATTGACGTCAATGACGGTAAATGGCCCGCCTGGCATTATGCCCAGTACATGACCTTATGGGACTTTCCTACTTGGCAGTACATCTACGTATTAGTCATCGCTATTACCATGGTGATGCGGTTTTGGCAGTACATCAATGGGCGTGGATAGCGGTTTGACTCACGGGGATTTCCAAGTCTCCACCCCATTGACGTCAATGGGAGTTTGTTTTGGCACCAAAATCAACGGGACTTTCCAAAATGTCGTAACAACTCCGCCCCATTGACGCAAATGGGCGGTAGGCGTGTACGGTGGGAGGTCTATATAAGCAGAGCTCGTTTAGTGAACCG

**Figure S1: UTR Linker sequences.** Sequence of the **A)** OpIE2-UTR-Linker and **B)** CMV-UTR-Linker. Components of the linkers are as follows: HDR (purple), SV40pA (blue), promoter (underlined).

**
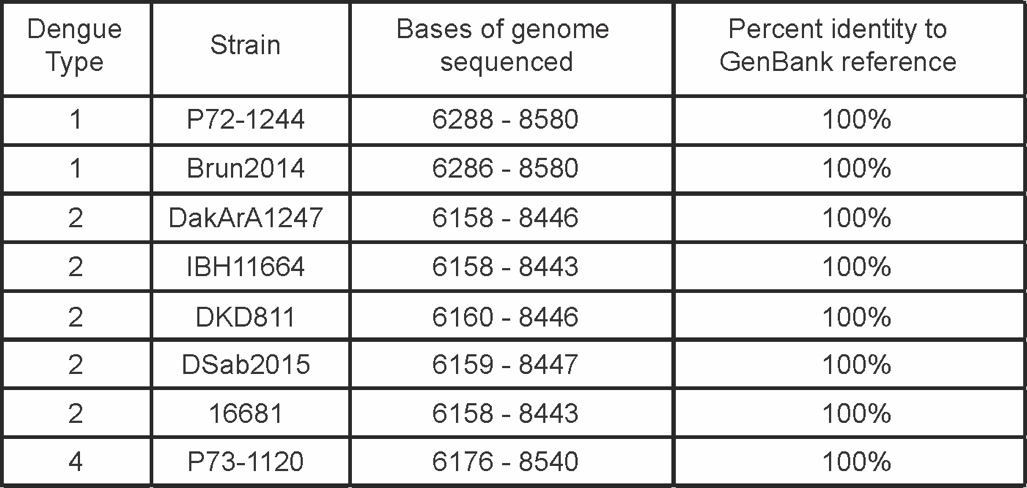
**

**Table S3: Sequencing results for passage 1 viruses generated in this paper.**

cDNA was synthesized from extracted viral RNA from passage 1 viruses generated in this paper. PCR amplification of the cDNA was performed using appropriate primers for the target sequences. The resulting PCR amplicons were purified sent for sequencing by Oxford Nanopore Technologies (ONT) via Plasmidsaurus.


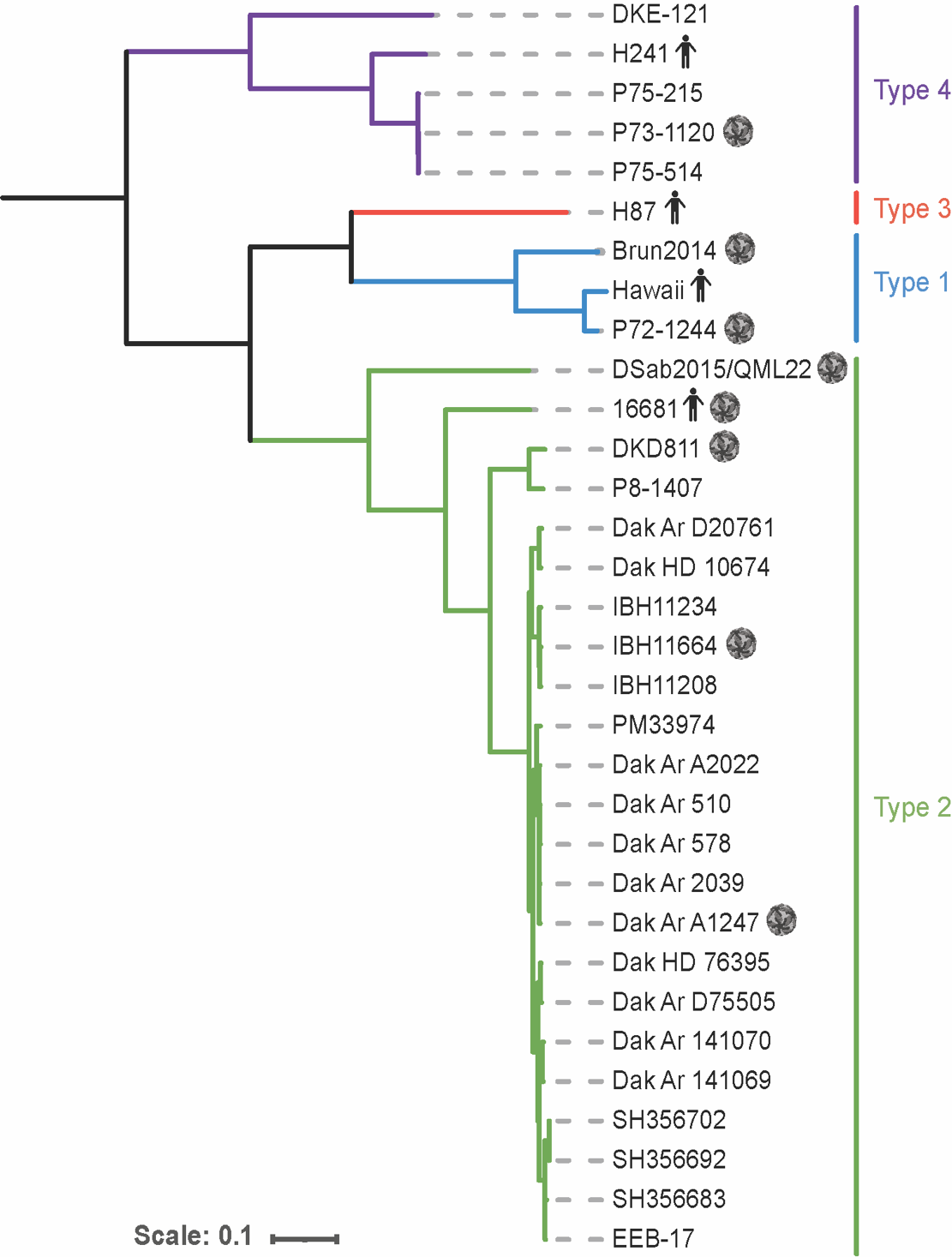


**Figure S2: Phylogeny of sylvatic dengue viruses with viruses rescued in this paper noted.** A full-length genome alignment of sylvatic dengue virus strains listed in Table 1, as well as 4 human dengue virus genomes (Type 1 – Hawaii [KM204119](https://www.ncbi.nlm.nih.gov/nuccore/KM204119); Type 2 – 16681 [NC_001474](https://www.ncbi.nlm.nih.gov/nuccore/NC_001474); Type 3 – H87 [M93130](https://www.ncbi.nlm.nih.gov/nuccore/M93130); Type 4 – H241 [AY947539](https://www.ncbi.nlm.nih.gov/nuccore/AY947539)) was created with the MEGA software (V10, <https://doi.org/10.1093/molbev/msz312>), using the Tamura-Nei substitution model and a Gamma distribution of 5. Aligned sequences were used to construct a maximum likelihood phylogenetic tree with the MEGA software (Version 10, <https://doi.org/10.1093/molbev/msz312>) using the Tamura-Nei substitution model and a Gamma distribution of 5. The resulting tree was visualized and edited using iTOL (<https://doi.org/10.1093/nar/gkae268>). Human dengue viruses are denoted with a human figure. Dengue viruses that were rescued in this study are denoted with a virus image next to the strain name.
